# Relationships between Plasma Micronutrients, Serum IgE, and Skin Test Reactivity and Asthma among School Children in Rural Southwest Nigeria

**DOI:** 10.1155/2014/106150

**Published:** 2014-05-25

**Authors:** Oluwafemi Oluwole, Olatunbosun G. Arinola, Mary D. Adu, Adedayo Adepoju, Babatunde O. Adedokun, Olufunmilayo I. Olopade, Christopher O. Olopade

**Affiliations:** ^1^Center for Clinical Cancer Genetics and the Center for Global Health, University of Chicago, Chicago, IL 60637, USA; ^2^College of Medicine, University of Ibadan, PMB 5017, Ibadan, Oyo State, Nigeria; ^3^Healthy Life for All Foundation, P.O. Box 3017, Ibadan, Oyo State, Nigeria; ^4^Section of Pulmonary and Critical Care, Department of Medicine and the Center for Global Health, University of Chicago, 5841 South Maryland Avenue, MC 6076, Chicago, IL 60637, USA

## Abstract

*Objective*. Increasing prevalence of asthma has been attributed to changes in lifestyle and environmental exposures. We conducted a case-control study to investigate the relationship between serum micronutrients and asthma in rural school children in Nigeria. *Methods*. We administered questionnaires to 1,562 children to identify children with asthma. Serum concentration levels of 12 micronutrients were determined in asthma cases (*N* = 37) and controls (*N* = 30). Allergy skin prick test and spirometry were also performed. *Results*. Plasma levels of the following micronutrients were significantly different between cases and controls: calcium (7.48 ± 2.16 versus 8.29 ± 1.62 mg/dL; *P* = 0.04), manganese (44.1 ± 11.5 versus 49.3 ± 7.9 mg/L; *P* = 0.01), selenium (76.1 ± 14.9 versus 63.3 ± 26.8 *μ*g/L; *P* = 0.02), and albumin (3.45 ± 0.90 versus 3.91 ± 0.99 g/dL; *P* = 0.04). Plasma concentrations of iron and selenium were positively correlated with lung function, *r* = 0.43 (*P* < 0.05 in each case) while manganese serum concentration was negatively correlated with asthma (*r* = −0.44; *P* < 0.05). *Conclusions*. Children with asthma had reduced levels of plasma manganese, calcium, and albumin but raised level of selenium. The protective or risk effects of these micronutrients on asthma warrant further investigation.

## 1. Introduction

In most developing countries, including Nigeria, asthma has become one of the most common chronic diseases among children. It is a major cause of emergency hospital visits and school absenteeism among children younger than 15 years of age in most developed countries [1, 2]. However, hospital visitations among children with asthma in Nigeria, especially in rural areas, remain very low due to limited access to healthcare services and increased focused efforts on diseases such as tuberculosis, measles, and malaria [3]. While asthma may be previously reported to be uncommon in most parts of sub-Saharan Africa [4, 5], recent studies have demonstrated a high prevalence of asthma and other respiratory diseases among school-aged children and adolescents in Nigeria [6–8]. Some population-based studies have attributed this increase to changes in lifestyles and nutrition, suggesting a likelihood of higher prevalence of asthma in countries where there is a shift from traditional to westernized lifestyles [9, 10].

Recent clinical observations and epidemiological studies have identified associations between nutritional elements (e.g., magnesium, calcium, copper, zinc, selenium, and vitamin D) and asthma prevalence [9, 11, 12]. It is thought that micronutrients influence the immune system and may play a major role in the development of asthma and in the progression of other allergic diseases [9, 13–15]. However, only few of these studies have used objectively measured variables to evaluate the relationships between nutrition and respiratory diseases [11, 16]. Low serum levels of certain micronutrients, besides causing other life-threatening consequences, may result in increased predisposition to asthma symptoms and allergic diseases in susceptible individuals [9, 17] due to profound effects on immune responses and oxidant-antioxidant balance.

Therefore, the search for causes of the increasing prevalence of asthma among children, especially in the rural communities in developing countries, may be linked to dietary factors. This link is based on the hypothesized benefits of antioxidant functions of certain micronutrients (selenium, zinc, and magnesium), which may modulate the amount of oxidants in the body [18, 19]. The resultant decrease in oxidative stress may be an important factor in the etiology of childhood asthma [18, 19].

Currently, among rural children with asthma in Nigeria, there are limited data on the relationships between asthma and allergy and the possible role of micronutrients. We conducted a population-based case-control pilot study to determine serum concentration levels of different nutritional micronutrients and to evaluate their relationships with asthma and allergy sensitization among school children in selected rural communities. In addition, we also evaluated the relationships of these micronutrients with lung function impairment.

## 2. Materials and Methods

The Institutional Review Board on Human and Animal Research at the University of Ibadan, Nigeria, and the University of Chicago, USA, approved the research protocol. Before starting the study, parents or guardians of all participants in the study provided written informed consents. Approval to conduct the study was also obtained from the administrators of participating schools and the Oyo State Ministry of Education and Youth Development, Ibadan, Nigeria.

### 2.1. Study Participants

The study was conducted in two phases in three rural communities: Abanla, Eruwa, and Igbo-Ora, all of which are located 40 to 100 km from Ibadan, a major urban center in Nigeria. The major occupation in these communities is farming. In Phase 1 of the study, 1,562 students aged 13 and 14 years were identified from the school registers of 20 high schools from the three rural communities. The age group of 13-14 years was selected in accordance with the research protocol of the International Study of Asthma and Allergies in Childhood (ISAAC) that used two age groups: 6-7 and 13-14 years of age [20]. Due to the pilot nature of the study, the older age group was used to minimize study cost and to recruit students who can complete the questionnaires on their own, thus eliminating the need to go into the communities to interview their parents or guardians. We administered the ISAAC questionnaires in English language to all the 1,562 students using a cross-sectional survey. However, 1,071 students self-completed and returned the questionnaires for a response rate of 69%. Children were screened for asthma based on the affirmative responses to the ISAAC questionnaire which includes questions on symptoms of asthma and/or physician's diagnosis of asthma. Subjects were deemed to have asthma if they were ever diagnosed with asthma by a physician, had experienced asthma-related symptoms such as wheezing or whistling in the chest in the last 12 months, or used asthma medication in the preceding year [21]. The specific ISAAC questionnaires include “Has a doctor ever told you that you have asthma?” Those that reported non-physician-diagnosed asthma were further assessed based on their “asthma-related symptom” if they reported a positive response to either “Have you ever had wheezing or whistling in the chest at any time in the past 12 months?” or “In the past 12 months have you had a dry cough at night, apart from a cough associated with a cold or chest infection?” Those that reported “No” responses to both questions on physician-diagnosed asthma and asthma-related symptoms were classified as children without asthma. Based on questionnaire responses and further medical examinations by a pediatric pulmonologist, 80 asthma cases were identified in Phase 1.

In Phase 2, asthma cases were matched to controls (children without asthma) based on age (within one year), gender, and school location. After obtaining written informed consent from the subjects and their parents/guardians, 37 asthma cases and 30 controls further consented to participate in the allergy skin testing and blood draw procedures, while only 25 cases and 21 controls from the 37 cases and 30 controls also consented to participate in pulmonary function evaluation. Exclusion criteria for the study included treatment with antihistamines or oral corticosteroids within three months, a history of smoking, or the presence of skin disease that may affect the results of the allergy skin testing. However, no subject was excluded on the basis of these preset criteria.

### 2.2. Sample Collection, Preparation, and Analytical Methods

A total volume of 10 mL venous blood samples were collected from the asthmatics and control subjects: 5 mL was collected in 5 mL Vacutainer EDTA tubes for hematological analysis, 4 mL in lithium heparin bottles for determination of trace elements, total proteins, and albumin, and 1 mL in plain bottles for the determination of IgE. Plasma was obtained from samples in lithium heparin bottles, while serum was separated from samples in plain bottles. The serum and plasma samples were frozen and stored at −80°C before measurement of IgE, total protein, albumin, globulin, and trace metals. All blood samples were processed and analyzed at the Institute for Advanced Medical Research and Training (IMRAT) and Immunology Laboratories at the University of Ibadan, Nigeria.

All of the materials (glass and plastic) used were thoroughly cleaned and rinsed with demineralized water. A complete blood count was performed using the Swelab 910EO+ autocounter (Swelab, Stockholm, Sweden), while the Swelab autoheater was used for the absolute eosinophil count. Total serum IgE levels (IU/mL) were measured in serum by the ELISA method, using the human IgE micro-ELISA test kit (Leinco Technologies Inc., St. Louis, MO). Total protein was determined using biuret reagent. Albumin was determined using immunoplates. Wells of the prepared immunoplates were filled with standard proteins (25%, 50%, 100%, and 200%) or plasma samples. The plates were incubated for 4 hours at room temperature and the diameters of precipitin rings were measured using an illuminated Hyland viewer with a micrometer eyepiece. The level of albumin in each sample was extrapolated from a standard graph. The concentration of albumin was subtracted from the concentration of total protein to determine the plasma levels of globulin. For plasma levels of trace metals, a Buck Scientific 205 flame atomic absorption spectrophotometer (AAS) (Buck Scientific, Connecticut, USA) was used.

### 2.3. Pulmonary Function Test

Pulmonary function tests were performed with the PC-based, full-function KoKo spirometer (Ferraris Respiratory, Louisville, Colorado) in accordance with the American Thoracic Society's (ATS) recommendation [22]. Spirometry was performed at similar times of the day to minimize diurnal variation. Predicted normal values for lung function variables were obtained from the ATS/ERS recommendations using NHANES reference equation which adjusts for sex, age, and height and serves as the most appropriate reference value for African populations [23]. The spirometer was calibrated daily and operated within the ambient temperature. Also, technicians had standardized training before starting the study. Short-acting inhaled bronchodilator therapy was withheld in children with asthma for at least six hours before spirometry testing. Subjects were tested for pulmonary function by completing spirometry maneuvers, to record forced vital capacity (FVC), forced expiratory volume in one second (FEV_1_), FEV_1_/FVC, forced expiratory flow between 25% and 75% of FVC (FEF_25–75%_), and peak expiratory flow rate (PEFR). The control subjects also had pulmonary function test performed. Children completed at least three, but not more than seven, maneuvers in a sitting position while wearing a nose clip. However, the best of 3 acceptable and reproducible efforts (with differences not more than 5% of each maneuver [22]) was used for analysis. To improve the diagnostic classification of asthma cases, a postbronchodilator test was also performed in which spirometry procedures were repeated 15 minutes after administering short-acting inhaled bronchodilator (albuterol). All spirometry tests were reviewed for quality control and assurance by an expert pulmonologist, and if judged inadequate, spirometry was repeated.

### 2.4. Allergy Skin Testing

An allergy skin test was performed using the Skintestor OMNI (Greer Laboratories, Inc., Lenoir, NC) on the volar side of the forearm with standardized extracts of the following eight allergens (Greer Laboratories): (1) mold mix (*Alternaria alternata, Aspergillus niger, Cladosporium sphaerospermum,* and* Penicillium notatum*), (2) house dust mite (*Dermatophagoides farinae*,* D. pteronyssinus*), (3) cat (*Felis catus domesticus*), (4) cockroach (*Blattella germanica, Periplaneta americana*), (5) dog (*Canis familiaris*), (6) mouse (*Mus musculus*), (7) mango (*Mangifera indica*), and (8) grass mix (Bermuda, Johnson, June, Meadow Fescue, Orchard, Red Top, Timothy, Sweet Vernal, Perennial, Rye, and 9 Southern Grass mix). Histamine was used as positive control while 50% gly  and  50% cocas were used as negative controls. Skin response was measured with a metered ruler after 15 minutes; wheal dimension of 3 mm or larger than the control was considered to be a positive reaction.

Atopy was defined as a positive skin test reaction to at least one of the applied allergens.

### 2.5. Data Analysis

Descriptive analyses were performed using mean, standard deviation, and counts based on distribution. The analysis for Phase 2 of the study was limited to the 67 (37 asthma cases and 30 nonasthma controls) participants, with complete information on micronutrients, allergy skin testing, and pulmonary function evaluation. Student's *t*-test was used to compare lung function parameters between the asthma cases and controls and a Wilcoxon rank-sum test was used to compare serum levels of micronutrients. Spearman's correlations were used to examine the relationship between variables. All statistical analyses were conducted with Stata 10.0 (College Station, TX). A *P* value less than or equal to 0.05 was considered statistically significant.

## 3. Results

### 3.1. Subjects and Asthma Prevalence

In total, one thousand and seventy-one (1,071) students from 20 high schools participated in Phase 1 of the study. The prevalence of asthma among the study population was 7.5% (80/1071, 95% confidence interval CI: 6.0–9.2%). The mean age of the students was 13.4 ± 0.5 years. Thirty-seven students (27 F and 10 M) with questionnaire responses consistent with asthma and 30 age-matched controls (24 F and 6 M) without asthma symptoms that had plasma micronutrients levels determined were included in Phase 2 of the study. Age, gender, and BMI were not significantly different between the cases and controls (Table 1).

### 3.2. Serum Micronutrient Concentrations

The mean plasma micronutrient values for asthma cases and controls together with the *P* values are presented in Table 2. The mean plasma concentration of selenium (76.1 ± 14.9 versus 63.3 ± 26.8 *μ*g/L) was higher, whereas the mean plasma concentrations of calcium 7.48 ± 2.16 versus 8.29 ± 1.62 mg/dL), manganese (44.1 ± 11.5 versus 49.3 ± 7.9 mg/L), and albumin (3.45 ± 0.90 versus 3.91 ± 0.99 g/dL) were significantly lower in cases relative to controls (*P* < 0.05). No significant differences were observed with the other micronutrients. In addition, serum levels of iron and selenium were positively correlated with FEV_1_/FVC (*r* = 0.43; *P* < 0.05 for both micronutrients), while serum levels of manganese were negatively correlated with asthma (*r* = −0.44; *P* < 0.05) (data not shown). *P* ^*^

### 3.3. Pulmonary Function Test

Of the 67 students enrolled, only 46 (25 asthmatic and 21 controls) were available for spirometry. Details of the pulmonary function tests are presented in Table 3. Although the peak expiratory flow rate was significantly lower among cases relative to controls (3.44 ± 1.19 versus 4.34 ± 1.49 PL/min; *P* < 0.05), all other spirometry variables were similar between cases and controls.

### 3.4. Relationship between Plasma Micronutrients, Total Serum IgE, Eosinophil Percentage, and Eosinophil Count

In the correlation analysis of pulmonary function parameters with micronutrient concentrations, only the plasma levels of iron and selenium were significantly associated with FEV_1_/FVC (*r* = 0.43, *P* < 0.05 in each case). Also, there was a negative correlation between serum IgE and plasma concentrations of copper, selenium, and albumin (*r* = −0.26, −0.29, and −0.30 resp., *P* < 0.05). Additionally, plasma concentration of cadmium showed a positive correlation with eosinophil percentage (*r* = 0.26, *P* < 0.05), while a significant negative correlation was also observed with serum copper (*r* = −0.34, *P* < 0.01). No significant correlation was observed between any of the plasma micronutrients and total eosinophil count. However, both copper and cadmium were significantly correlated with eosinophil percentage (*r* = −0.34 and 0.26, resp.) (Table 4). The results of the relationship between plasma micronutrients and measures of allergic skin tests are shown in Table 5. Only the plasma concentration of manganese was significantly related to a positive skin test in response to cockroach, mold, dust mite, and pollen antigens (*r* = −0.24, −0.38, −0.25, and −0.25 resp., *P* < 0.05). Serum magnesium was also significantly associated with positive skin reactivity to mold (*r* = −0.28, *P* < 0.05). Although the above relationships were statistically significant, they were weak relationships.

## 4. Discussion

In this pilot study, asthma prevalence was observed to be 7.5% among 13- to 14-year-old school children from three rural communities. This is slightly higher than 7.2% reported in 2004 among children in a major urban center (Ibadan) within the same geographical region [8]. This suggests that while asthma may be previously thought to be uncommon in Nigeria, especially in the rural areas, there is a gradual increase in the prevalence of asthma and the urban-rural prevalence gradient may be disappearing. Since rural children in Nigeria may have poor nutrition and antioxidant defense system [24], it is necessary to assess the relationships between nutritional micronutrients and asthma. Our study showed that children with asthma had significantly lower mean calcium, manganese, and albumin levels when compared to controls. It was also observed that plasma levels of iron and selenium were positively correlated with lung function (*r* = 0.43; *P* < 0.05), while manganese serum level was negatively correlated with lung function (*r* = −0.44;  *P* < 0.05) (data not shown).

Significantly lower calcium, manganese, and albumin levels were found in children with asthma, while normal serum zinc levels were observed in both cases and controls. Although the zinc levels were normal in both groups, low zinc levels may be associated with the risk of developing asthma [25, 26], as zinc replacement therapy has been shown to be beneficial in asthma treatment [27–29]. Additionally, low plasma zinc levels observed during an acute asthma exacerbation have been described to increase the clinical manifestation of the disease, due to increased oxidant stress, resulting in inflammation and hyperreactivity in the airway [30]. Therefore, dietary zinc supplementations in children may be a plausible recommendation for immune modulation to reduce the risk and severity of acute respiratory infection [31]. Significantly lower albumin has also been reported in asthmatic children in other studies [32].

Diet may influence the prevalence of asthma in three distinct ways: food allergens that can cause asthma; breast-feeding for the prevention of asthma later in life [33–36]; and through low intake of antioxidants [11, 13, 14]. The growing understanding of the role of free oxygen radicals in the pathogenesis of asthma has drawn particular attention to the function of antioxidant defense systems and selenium. Selenium functions as a cofactor for the antioxidant anti-inflammatory enzyme, glutathione peroxidase, which protects the lungs from the potentially damaging effects of free oxygen radicals [30, 37–40]. The antioxidant status may affect asthma risk by influencing the development of asthmatic immune phenotype, modulating the asthmatic response to antigen provocation, or affecting the inflammatory response during and after an asthma attack [13]. Many epidemiological studies have associated low selenium levels with the development and manifestation of asthma [41–44] through reduced glutathione peroxidase (GSHPx) activity [45–47]. One study linked a mean increase in selenium serum levels (OR = 0.88; 95% CI: 0.7–1.1) to a 10–20% reduction in asthma prevalence in children aged 4 to 16 years [13].

Recent population studies on selenium supplementation in the diet of adults in the United Kingdom and in a conglomerate of 14 European countries did not demonstrate a protective effect from asthma [48, 49]. Another randomized controlled trial in which selenium was supplemented in the diet of asthmatics also failed to demonstrate beneficial effects [47]. While there are limitations to the design and conduct of these studies, these investigations indicate that the role of selenium in modulating asthma remains unclear. In our study, which involved a much younger cohort of children with mild and stable asthma, it was observed that the selenium levels in the 2 groups were within normal reference range but were significantly higher in the asthmatics relative to the controls. The clinical implication of this observation is unclear, as there was no information on the dietary intake for each group. It is possible that the effects of selenium may be more beneficial in a population exhibiting more severe asthma, an avenue of future research in a larger study. In addition, further studies could also estimate the dietary intake of micronutrients in the participants.

Findings from this study also indicate that magnesium may be associated with atopy in children with asthma. A low serum level of magnesium has been previously associated with asthma symptoms in adult Nigerian asthmatic patients [50]. Epidemiological evidence from population-based studies also indicates that magnesium has a bronchodilating effect [51] and may play a beneficial role in the development of asthma [45, 46, 50–52]. A deficiency of magnesium may be associated with increased incidence of brittle asthma, wheezing, and reduced lung function [53–59]. Low magnesium stores in the body enhance intracellular influx of calcium, which increases the contractility of bronchial smooth muscle cells [52]. As the contraction of smooth muscle cells is important in the development of asthma, a magnesium deficiency might adversely influence asthma control [60–62]. In addition, patients with severe asthma have been reported to have low serum magnesium levels (serum level <0.74 mmol/L) and a higher incidence of asthma exacerbation and hospitalization than asthmatic patients with normal serum magnesium levels [63, 64].

Other studies have asserted that a higher dietary intake of magnesium is significantly associated with reduced bronchial hyperreactivity and wheezing [15, 53], while low intake is associated with lower lung function and comprises a significant risk factor for reported wheezy illness among children [56, 65, 66]. While magnesium supplementation in patients suffering from acute asthma has produced beneficial outcomes [53, 56], there are also inconsistencies in its protective effects for asthma. In this study, the serum concentration of magnesium was similar in both asthma cases and controls. It was observed, however, that the magnesium level was positively correlated with an atopic tendency to mold allergens (*r* = 0.28; *P* < 0.05). This observation is consistent with findings of Gontijo-Amaral et al. [67], who observed that magnesium supplementation decreased bronchial reactivity and positive skin reaction to recognized antigens, suggesting the potential beneficial effects of magnesium in asthma. Further large-scale studies are necessary to better define the potential role of magnesium in the prevalence and treatment of asthma in children.

The literature suggests inconsistent roles for many serum micronutrients, which may lead to the suggestion that these results may reflect random variations that are unrelated to micronutrient levels or effects [52, 68]. For example, among other factors being investigated in the global increase in asthma prevalence is the impact of changing diet. There is interest in the suggestion that changes in maternal diet during pregnancy and diet during early childhood may influence the risk of developing asthma [69, 70]. The selenium status of mothers during early pregnancy was found to be associated with early childhood wheezing but not with asthma or atopic sensitization. This association was absent beyond the age of 5 years [70].

Animal studies of diet supplementation with low, medium, and high doses of selenium before ovalbumin challenge show that selenium intake and allergic airway inflammation are not related to dosage level [71]. This phenomenon may explain the inconsistent results in human studies involving selenium supplementation. In addition, it has been suggested that the effect of dietary factors is evidenced primarily on the development of asthma and allergy during the crucial period of fetal development, when a slight perturbation in organogenesis can exert a disproportionately potent effect [33]. This trend could possibly explain the ineffectiveness of dietary supplementation later in life and the inconsistent associations that have been reported for many cross-sectional studies of older children and adults.

As the effects of diet on asthma may be due to specific nutrients, specific food, or a specific period of early development, studying the role of individual micronutrient intake is relevant to the understanding of the biological mechanisms behind the observed association. Information is also needed on whether specific micronutrient intake or food has an independent effect on respiratory disease or whether the overall effect of different micronutrients is larger than the independent effects due to interaction between dietary components. The inconsistencies in reports of the beneficial effects of micronutrients in many studies may be as a result of differences in the methods of data collection and analysis, the existence of variability in the relationship between diet and respiratory disease across population, and the bias in reporting and publishing more frequently statistical significant findings than negative findings.

We acknowledge that the study has some limitations. First, while a case-control design was incorporated, the data was collected at one point in time and used prevalent cases, which makes the study vulnerable to cross-sectional limitations, including a lack of temporal assessment. However, due to practical considerations, this model has been typical of asthma case-control studies [72, 73]. The lack of statistical significant differences in many of the serum micronutrients between asthma cases and controls might have resulted from the study being underpowered due to the small number of subjects enrolled in the study, resulting in a type 1 error. This is because, in many rural areas in Nigeria, asthma still remains relatively uncommon. In addition to limited access to healthcare services in rural areas, which may result in asthma being underdiagnosed, the low asthma prevalence may be due to the identified protective effects of farming and rural living as suggested by the “hygiene hypotheses.” Due to many other logistic problems associated with conducting studies in rural areas in developing countries, we were unable to screen a much larger population of school children to increase the possibility of detecting more asthma cases in this population of rural school children. Finally, history of dietary intake in the sampled children was not studied. The relationships between asthma and dietary intake of micronutrients have been established in studies investigating prevalence of wheeze and bronchial hyperresponsiveness (BHR) [74, 75]. Therefore, if the important question is to know whether serum micronutrients influence the development and progression of asthma, it may seem more logical to investigate the overall pattern of nutritional status (measured as indices of dietary intake and serum nutritional biomarkers) and its association with asthma and lung function outcomes.

## 5. Conclusion

This study contributes to the growing body of literature indicating that children with higher serum manganese, calcium, and albumin concentrations are less likely to have asthma. While studies investigating possible relationships between selenium levels and asthma have yielded contradictory results, this study also found a significantly higher concentration of selenium in asthmatic children compared to controls, suggesting that the protective effect of selenium against asthma warrants further investigation. For the first time in Nigerian scientific literature, this study demonstrates that lung function in stable asthmatics may not be influenced by levels of micronutrients. Further research is needed to elucidate the mechanisms behind the complex relationships found in this study and to investigate the associations between nutrition and augmented antioxidant defense system and their influences on asthma development and exacerbations.

## Figures and Tables

**Table 1:** Characteristics of cases and controls.

	Cases (*n* = 37)	Controls (*n* = 30)	*P* value
Age (years)			0.06∗
13	16 (43.2%)	21 (70.0%)	
14	21 (56.8%)	9 (30.0%)	
Gender			0.57∗
Male	27 (73.0%)	24 (80.0%)	
Female	10 (27.0%)	6 (20.0%)	
BMI (±SD)	17.4 ± 2.9	17.0 ± 2.1	0.13

^*^ *P* values are for overall trend.

**Table 2:** Serum proteins and micronutrients in asthma cases and controls.

Micronutrient (normal range)	Asthma cases (*n* = 37)	Controls (*n* = 30)	*P* ^*^
	Mean ± SD	Mean ± SD	
Magnesium (18–30 mg/L)	33.7 ± 3.8	33.1 ± 4.5	0.49
Zinc (65–145 *μ*g/mL)	100.6 ± 22.8	106.3 ± 24.7	0.44
Calcium (8.5–10.5 mg/dL)	7.48 ± 2.16	8.29 ± 1.62	0.04
Iron (60–175 *μ*g/mL)	78.2 ± 14.9	72.7 ± 22.4	0.17
Manganese (35–100 *μ*g/L)	44.1 ± 11.5	49.3 ± 7.9	0.01
Copper (70–133 *μ*g/mL)	50.2 ± 8.9	53.3 ± 9.5	0.11
Cadmium (30–300 *μ*g/dL)	36.9 ± 6.4	35.9 ± 5.1	0.78
Chromium (≤140 *μ*g/dL)	35.1 ± 6.3	34.2 ± 5.3	0.96
Selenium (80–280 *μ*g/L)	76.1 ± 14.9	63.3 ± 26.8	0.02
Total protein (5.0–8.0 g/dL)	7.28 ± 0.48	7.28 ± 0.73	0.84
Albumin (3.0–5.0 g/dL)	3.45 ± 0.90	3.91 ± 0.99	0.04
Globulin (2.0–3.0 g/dL)	3.79 ± 1.10	3.37 ± 0.98	0.10

^*^ *P* values were calculated from the Wilcoxon rank-sum test. The pair-ship was ignored because only 16 matched pairs have data in the two matched individuals.

**Table 3:** Pulmonary function test in asthma cases and controls.

Pre-BD	Cases (*n* = 25)	Controls (*n* = 21)	*P* ^*^ value
	Mean ± SD	Mean ± SD	
FVC, L	2.45 ± 0.70	2.54 ± 0.86	n.s
FEV_1_, L	1.92 ± 0.34	1.96 ± 0.53	n.s
FEV_1_, % of predicted	75 ± 10	77 ± 19	n.s
FEV_1_/FVC	0.82 ± 0.18	0.82 ± 0.22	n.s
PEFR, L/S	3.44 ± 1.19	4.34 ± 1.49	0.03

^*^ *P* values were calculated from *t*-tests; n.s: Not significant.

**Table 4:** Spearman's correlation (*r*) between serum micronutrients, serum IgE, eosinophil count, and eosinophil percentage.

Micronutrient	Serum IgE	Eosinophil count	Eosinophil %
Magnesium	0.14	−0.07	0.11
Zinc	0.01	0.03	−0.10
Calcium	−0.20	−0.03	−0.19
Iron	0.22	0.13	−0.03
Manganese	−0.21	−0.16	−0.33
Copper	−0.26∗	−0.16	−0.34∗∗
Cadmium	0.11	0.09	0.26∗
Chromium	0.08	0.03	0.19
Selenium	0.29∗	0.25	0.13
Total protein	−0.07	−0.14	−0.10
Albumin	−0.30∗	−0.09	−0.02
Globulin	0.19	0.01	−0.03

^*^ *P* < 0.05, ∗∗*P* < 0.01.

**Table 5:** Spearman's correlation (*r*) between serum micronutrient concentrations and allergy skin test.

Micronutrient	Stand. cat hair	Dog epith. mixed breed	GS9 southern grass mix	GS2 cockroach mix	Mango blossom	Stand. mite	GS mold mix number 3	Mouse epith.
Magnesium	0.09	0.04	0.07	0.01	0.09	0.20	0.28∗	0.01
Zinc	−0.15	−0.20	−0.11	0.04	0.003	0.03	−0.10	−0.04
Calcium	−0.11	−0.06	−0.13	0.001	0.02	0.11	−0.12	0.10
Iron	−0.03	0.03	−0.003	−0.01	0.03	0.11	−0.02	0.15
Manganese	−0.18	−0.20	−0.25∗	−0.24∗	−0.20	−0.25∗	−0.38∗∗	−0.15
Copper	−0.19	0.02	−0.07	−0.12	−0.09	−0.14	−0.11	0.03
Cadmium	0.09	0.05	0.07	0.008	0.08	0.10	0.21	0.14
Chromium	0.07	0.01	0.06	0.001	0.06	0.03	0.14	0.05
Selenium	0.03	0.02	0.04	0.16	0.09	0.05	−0.07	0.08
Total protein	0.03	0.11	0.09	0.02	−0.02	0.06	0.03	−0.05
Albumin	−0.05	0.04	−0.07	0.00	0.07	0.10	0.07	0.07
Globulin	0.08	0.05	0.13	0.03	−0.03	−0.04	0.005	−0.06

^*^ *P* < 0.05, ∗∗*P* < 0.01.
